# Financial conflicts of interest among authors of clinical practice guidelines for diabetes mellitus in Japan

**DOI:** 10.1111/1753-0407.13533

**Published:** 2024-04-10

**Authors:** Anju Murayama

**Affiliations:** ^1^ Tohoku University School of Medicine Sendai Japan; ^2^ Department of Population Health Science and Policy Icahn School of Medicine at Mount Sinai New York New York USA

Clinical practice guidelines (CPGs) serve as pivotal frameworks for standardizing evidence‐based diagnostic and therapeutic approaches, particularly in the management of diabetes and beyond.^1^, ^2^ However, the integrity of these guidelines can be compromised by conflicts of interest (COIs).^3^, ^4^, ^5^ Given that current increasing attention from pharmaceutical companies to diabetologists^6^, ^7^ and large prevalence of diabetes and obesity, proper management of financial COIs is essential for trustworthy diabetes CPGs.^1^ Despite the critical nature of this issue, no research has investigated these financial relationships in the Japanese context.

Using a publicly accessible database (https://yenfordocs.jp/) containing personal payments for lecturing, consulting, and manuscript drafting from all pharmaceutical companies affiliated with the Japan Pharmaceutical Manufacturers Association, this study examined personal payments made to all authors for Japanese Clinical Practice Guideline for Diabetes 2019 (JCPGD) developed by the Japan Diabetes Society in 2019.^8^ Descriptive analysis was performed on the payment data extracted from the database between 2016 and 2020.

Among all 135 JCPGD authors, 129 (95.6%) received at least one personal payment for lecturing, consulting, and manuscript drafting from the pharmaceutical companies over the 5 years (Table 1). A total of 19 755 payments, amounting to $23 130 423, were made to the JCPGD authors by the pharmaceutical companies. The median payments per author were $89 955 (interquartile range: $7954–$258 527). More than 74.1% (100 authors), 60.7% (82 authors), and 47.4% (64 authors) received more than $10 000, $50 000, and $100 000 in total payments over the 5 years, respectively. The JCPGD chairperson received $207 889 before the JCPGD publication (2016–2018).

**TABLE 1:** Summary of personal payments from pharmaceutical companies to the authors of Japanese Clinical Practice Guideline for Diabetes 2019.

Variables	2016	2017	2018	2019	2020	Value
Total amount of payments, $	5 159 566	5 329 700	4 864 444	4 749 531	3 027 182	23 130 423
Mean payments per author (SD), $	38 219 (50263)	39 479 (50816)	36 033 (47588)	35 182 (46624)	22 424 (33331)	171 336 (220502)
Median payments per author (interquartile range), $	15 478 (1598–60 908)	19 501 (1251–57 136)	15 821 (2271–53 604)	15 802 (1284–52 045)	7571 (546–32 581)	89 955 (7954–258 527)
Range, $	0–271 491	0–232 162	0–245 168	0–216 321	0–210 167	0–1 087 444
Authors with payments, *n* (%)						
Any payments	120 (88.9)	117 (86.7)	115 (85.2)	119 (88.2)	106 (78.5)	129 (95.6)
>$10 000	79 (58.5)	82 (60.7)	82 (60.7)	77 (57.0)	63 (46.7)	100 (74.1)
>$50 000	38 (28.2)	41 (30.4)	37 (27.4)	35 (35.9)	23 (17.0)	82 (60.7)
>$100 000	15 (11.1)	15 (11.1)	11 (8.2)	13 (9.6)	3 (2.2)	64 (47.4)
>$250 000	1 (0.7)	0 (0)	0 (0)	0 (0)	0 (0)	36 (26.7)
>$500 000	0 (0)	0 (0)	0 (0)	0 (0)	0 (0)	12 (8.9)

*Note*: Japanese yen (¥) were converted to US dollars ($) using the 2020 average monthly exchange rate of ¥106.8 per $1.

Of 135 authors, 80 (59.3%) self‐declared financial COIs with companies between 2016 and 2018. However, the Japan Diabetes Society allowed the CPG authors to omit declaring financial COIs below a certain monetary threshold (eg, 500 000 Japanese yen, equivalent to $4683, or more per year per company for lecturing, honoraria, and drafting compensations). Consequently, 55 (40.7%) authors declared no COIs between 2016 and 2018, although 87.2% (48 out of 55) of these authors received at least some personal payments during the declaration period (2016–2018).

This study examined the size and prevalence of financial conflicts of interest among authors of the JCPGD 2019. Surprisingly, more than 95% of the JCPGD authors received more than $23.1 million in personal payments from pharmaceutical companies. Furthermore, the chairpersons received considerable amounts of personal payments during the guideline development period. The high percentage of JCPGD authors with financial COIs, the chairpersons' receipt of personal payments, and limited COI declarations are clear deviations from current international COI management policies.^1^, ^9^, ^10^ However, these findings were consistent with previous research in Japan^11^, ^12^, ^13^, ^14^, ^15^, ^16^, ^17^, ^18^, ^19^ and highlight the urgent need for substantial improvement in COI management strategies among CPG authors in Japan in the field of diabetology and endocrinology.

This study has several limitations. The study design precludes longitudinal analysis, and the focus on a single set of guidelines may not be generalizable to other fields or countries. Additionally, as the payment data were only voluntarily disclosed by companies belonging to the Japan Pharmaceutical Manufacturers Association, there could be unmeasured financial relationships between the JCPGD authors and undisclosed pharmaceutical companies.

## AUTHOR CONTRIBUTIONS

Anju Murayama: conceptualization; methodology; resource; software; formal analysis; investigation; writing—original draft; writing—review & editing; visualization; study administration.

## FUNDING INFORMATION

The author declares that there were no funding sources for this study.

## CONFLICT OF INTEREST STATEMENT

The author declares that there were no conflicts of interest for this study.

## INSTITUTIONAL BOARD REVIEW STATEMENT

As this study was a retrospective analysis of publicly available data and met the definition of nonhuman subjects research, no institutional board review and approval were required. This study followed the Strengthening the Reporting of Observational Studies in Epidemiology (STROBE) guideline.

## Data Availability

All data used in this study are available from the Japan Diabetes Society (http://www.jds.or.jp/modules/publication/index.php?content_id=4), Yen For Docs database maintained by Medical Governance Research Institute (https://yenfordocs.jp/), and pharmaceutical companies belonging to the Japan Pharmaceutical Manufacturers Association.
